# Goodnight book: sleep consolidation improves word learning via storybooks

**DOI:** 10.3389/fpsyg.2014.00184

**Published:** 2014-03-04

**Authors:** Sophie E. Williams, Jessica S. Horst

**Affiliations:** School of Psychology, University of SussexBrighton, UK

**Keywords:** word learning, sleep, shared storybook reading, language acquisition, memory consolidation

## Abstract

Reading the same storybooks repeatedly helps preschool children learn words. In addition, sleeping shortly after learning also facilitates memory consolidation and aids learning in older children and adults. The current study explored how sleep promotes word learning in preschool children using a shared storybook reading task. Children were either read the same story repeatedly or different stories and either napped after the stories or remained awake. Children's word retention were tested 2.5 h later, 24 h later, and 7 days later. Results demonstrate strong, persistent effects for both repeated readings and sleep consolidation on young children's word learning. A key finding is that children who read different stories before napping learned words as well as children who had the advantage of hearing the same story. In contrast, children who read different stories and remained awake never caught up to their peers on later word learning tests. Implications for educational practices are discussed.

## Introduction

Young children frequently ask for a favorite story to be read repeatedly (Sulzby, 1985)—particularly at bedtime (Sénéchal and LeFevre, 2001; Burke et al., 2004). This may be highly beneficial because repeatedly reading the same stories facilitates word learning (Sénéchal, 1997; Horst et al., 2011; McLeod and McDade, 2011; Wilkinson and Houston-Price, 2013) and reading stories can reduce the length of the bedtime routine (Field and Hernandez-Reif, 2001). Recent research also demonstrates a profound effect of sleep consolidation on word recall in adults (e.g., Dumay and Gaskell, 2012) and school-aged children (e.g., Gais et al., 2006; Brown et al., 2012). In the current study we explore how shared storybook reading immediately before a period of sleep facilitates preschool children's word learning.

### Shared storybook reading

Shared storybook reading helps young children learn new vocabulary (Hargrave and Sénéchal, 2000; Reese et al., 2010) and promotes later academic success (Whitehurst et al., 1988; Rimm-Kaufman and Pianta, 2000). Preschool children especially benefit when the same stories are read repeatedly (Sénéchal, 1997; Horst et al., 2011; McLeod and McDade, 2011). For example, Sénéchal (1997) tested children either after a single reading of a storybook or after repeated readings of the same storybook. Repeated readings increased both expressive and receptive word learning. Recently, McLeod and McDade (2011) explored the effects of repeated readings as well as contextual diversity. Children were tested in one of two conditions. In one condition, children heard a storybook, which contained each novel word once, read three times. In the other condition, children heard a storybook, which contained each novel word in three different contexts, read once. Children who heard the same story repeatedly demonstrated significantly better word learning than children who heard the diverse storybook once. Taken together, these studies demonstrate a clear advantage for reading stories repeatedly. However, the strength of this advantage remains unclear due to the methodological differences between conditions. For example, the amount of time children spent engaged in reading was less for children who only heard one story (see also Horst, 2013, for further review of methodological concerns).

In another recent study, overall storybook exposure was experimentally controlled by reading children either the same stories repeatedly or different stories (Horst et al., 2011). All children heard three stories during each session and had the same exposure to the novel words embedded within the stories. The only difference between conditions was whether the story context remained the same for the three readings or changed with each story reading. Children in the same stories condition learned significantly more novel words over the course of 1 week than children in the different stories condition. The authors argued that children learned more words when read the same stories repeatedly because such contextual repetition reduces the cognitive demands of the task, which, in turn, leads to better long-term learning (see also Horst, 2013).

To further test this explanation, Williams et al. (2011) also read children either the same or different stories; however, they increased the difficulty of the repeated readings condition by repeating the stories across days. Children in both conditions heard three different stories during each session over the course of 1 week. Here the only difference between groups was whether the same three stories were read during each session or whether three new stories were read during each session. Despite increasing the difficulty, children in the same stories condition learned significantly more novel words than children in the different stories condition.

Horst (2013) has argued that children in these studies, as well as others (e.g., Ard and Beverly, 2004; McLeod and McDade, 2011), benefited from contextual repetition, which lowers the cognitive demands of the word learning task and consequently frees up cognitive resources to facilitate encoding of new information. However, encoding is only one stage of memory processing (Diekelmann et al., 2009; Robertson, 2009). For robust word learning to occur, children must also consolidate the new information and retrieve it after a delay (Horst and Samuelson, 2008).

### Sleep consolidation

Sleep is a powerful aid in memory consolidation (see Diekelmann et al., 2009, for a review), allowing children and adults to better recall newly encoded information at a later time (Wilhelm et al., 2013). Sleep supports many cognitive functions including learning object locations (Kurdziel et al., 2013), relationships among objects (Lau et al., 2010), and face processing (Mograss et al., 2006). In particular, sleep supports the consolidation of declarative memory (see Ellenbogen et al., 2006, for a review)—the kind of memory involved in recalling new words (Robertson, 2009).

Sleep is most effective if it follows within a few hours of learning to reduce interference of the memory traces (Gais et al., 2006; Diekelmann et al., 2009). Even short naps provide beneficial effects of memory encoding. For example, Lahl et al. (2008) gave adults lists of adjectives to learn before napping or an equivalent period awake. Adults remembered words significantly better after an ultra short nap of only 6 min than after remaining awake for the same amount of time. However, napping for approximately 30 min promoted even better learning.

Naps also facilitate early language acquisition, particularly abstraction (e.g., learning one element predicts another later element as in “See the *cars*? Do you like *them*?”). For example, Gómez et al. (2006) exposed 15-month-old toddlers to an artificial language for 15 min at home before they napped or remained awake. When tested 4 h later in the lab, toddlers who had slept demonstrated an understanding of the abstract structure of the language, but the toddlers who remained awake did not, indicating sleep facilitated abstraction. However, another possible explanation is that toddlers who napped were simply better rested at test. In a follow-up experiment toddlers were exposed to the same language before a regular nap time and tested 24 h later (Hupbach et al., 2009). Again, when toddlers napped shortly after exposure to the language, they learned the general abstract structure, suggesting the original effect found by Gómez et al. (2006) was due to sleep consolidation and not simply being well-rested at test. In another condition, toddlers were familiarized to the artificial language at least 4 h before their next nap and tested 24 h later (Hupbach et al., 2009). When toddlers did not nap shortly after the learning phase they did not learn the abstract structure of the language, suggesting that the benefits of sleep consolidation are strongest if sleep follows shortly after learning (see also Gais et al., 2006; Diekelmann et al., 2009).

Work by Gaskell and colleagues (Dumay and Gaskell, 2007, 2012; Brown et al., 2012; Henderson et al., 2012) also demonstrates a benefit of sleep consolidation on language processing (see also Backhaus et al., 2008). For example, adults incorporate novel pseudo-words into their existing lexicons better if they learn the words in the evening prior to sleeping than if they learn the words in the morning (Dumay and Gaskell, 2007, 2012). A similar result has been found with 9-year-old children (Henderson et al., 2012). In this case, children were randomly assigned to learn new pseudo-words in the early morning or late afternoon. Children who learned the words in the evening prior to sleeping performed significantly better on cued word recall tests and continued to perform well the next day and 1 week later. Children who learned the words in the morning only performed well after they had had their overnight sleep, and then also continued to perform well 1 week later.

A similar effect has also been found by Backhaus et al. (2008) who trained 9–12-year-old children on lists of noun pairs both in the evening before sleep and in the morning. When children learned the words in the evening, they were significantly better at cued recall on both retention tests (the next morning and the next evening) than when they learned the words in the morning. In both conditions, children's performance improved following a period of sleep. That is, when children learned the list before a period of wakefulness, their recall did improve after their normal overnight sleep. Similarly, 7-year-old children are significantly more accurate on cued recall tests of newly learned pseudo-words after a longer retention interval including a period of overnight sleep than after a shorter retention interval of only 3–4 h that does not include sleep (Brown et al., 2012). Taken together, these studies present compelling evidence that sleep promotes memory consolidation in word learning studies for both older children and adults.

### The current study

In the current study we explored how sleep promotes word learning in preschool children using a shared storybook reading task. Half of the children habitually took afternoon naps and half of the children did not. Note that preschool children who habitually nap and those who do not habitually nap sleep for equivalent amounts of time within 24-h periods because those who do not nap sleep for longer at night (Ward et al., 2008; Lam et al., 2011). In addition, children were either read the same story three times or were read three different stories (for a similar method see Horst et al., 2011). Each story contained two novel name-object pairs and all children received the same exposure to each name-object pair (this is in line with the number of words children this age can learn within a given day, see Bion et al., 2013, and the number of words children can learn from storybooks, see Biemiller and Boote, 2006). Children's word learning was tested immediately, after their naps (nap conditions) or after the same amount of time awake (no nap conditions), as well as after their regular overnight sleep (24 h later) and after 7 days. To extend the previous research on repeated readings, we also included a ratings task to better understand the impact of repeated readings on children's enjoyment. Finally, we included plot comprehension questions as a control to ensure children were listening to the stories.

Based on previous research (e.g., Horst et al., 2011; Williams et al., 2011; Wilkinson and Houston-Price, 2013), we expect that children in the same stories conditions will demonstrate better word learning than children in the different stories conditions. Importantly, if sleep consolidation promotes word learning, then children who nap after hearing the stories should perform better than children who do not nap and performance should generally improve after overnight sleep. A critical test for the benefit of sleep consolidation on word learning will be the performance of the children who hear different stories and then nap. Learning words from different stories is challenging (e.g., Horst et al., 2011); however, sleep consolidation is highly effective if it occurs shortly after learning (Gais et al., 2006; Diekelmann et al., 2009; Hupbach et al., 2009). If sleep consolidation has a strong influence on word learning, then these children should later perform at levels similar to children who had the advantage of hearing the same story repeatedly. In contrast, if sleep consolidation has little influence on word learning, then both groups of children who hear different stories should perform similarly and we should find no effect of sleep.

## Methods

### Participants

Forty-eight 3-year-old children participated. Children were monolingual, British English speakers from primarily white, middle-class families living in an urban area on the English Channel and had no known learning difficulties. Children were recruited through nurseries and preschools. As a thank you, nurseries and pre-schools received book tokens and each child received several stickers. An additional four children were tested but their data not included in the final sample because they failed to cooperate (*n* = 1) or missed the final test due to absence (*n* = 3).

Children were quasi-randomly assigned to four conditions based on whether or not they habitually napped. Half of the children were read the same story and half were read different stories. This resulted in the following groups: same story nap (8 girls, 4 boys, *M* = 42 months, 6 days, *SD* = 2 months, 20 days), same story no nap (5 girls, 7 boys, *M* = 41 months, 26 days, *SD* = 3 months, 14 days), different stories nap (8 girls, 4 boys, *M* = 42 months, 1 day, *SD* = 2 months, 10 days), and different stories no nap (6 girls, 6 boys, *M* = 43 months, 14 days, *SD* = 3 months, 9 days). There was no difference in age between groups, *F*_(3, 44)_ = 0.71, *p* = 0.55.

### Stimuli

Children were read either one or three short storybooks minimally modified from those created by Horst et al. (2011): *Rosie's Bad Baking Day, The Very Naughty Puppy* and *Nosy Rosie at the Restaurant.* All three stories were compiled into one spiral-bound covered book where they appeared as chapters. For more information on the storybooks see Horst et al. (2011). Throughout each story, two novel objects were each depicted and named four times but were not the focus of the plot: an inverted slingshot that functioned like a hand mixer (*sprock*) and a kinetic wheel that functioned like a rolling pin (*tannin*). The objects appeared twice on their own pages and twice together.

#### Test stimuli

To test whether children learned the target words, an A4 spiral-bound test booklet with three practice pages and 13 test pages was used. Practice pages included pictures of four different familiar objects (e.g., ball, fish, plane, and car) and test pages included pictures of four novel objects (*M* = 4.07 × 6.43 cm *SD* = 1.25 cm). Throughout the test pages the novel targets (*sprock*, *tannin*) appeared both individually and together. The other novel objects were novel distractors that the children had not previously seen (see also, Werchan and Gómez, 2014). Picture locations (e.g., top left) were counterbalanced across pages.

### Procedure and design

**Figure 1:**
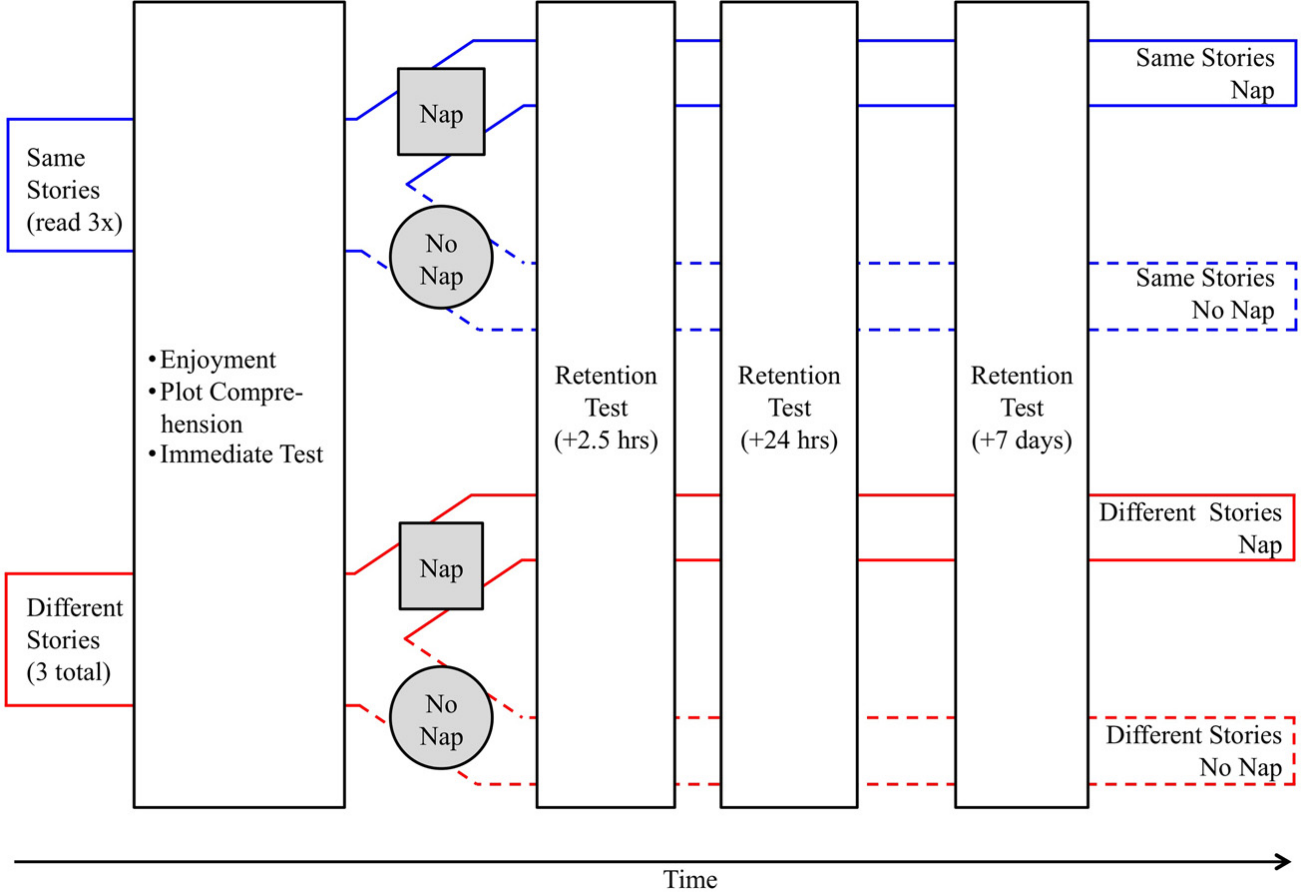
**Schematic representation of the experimental design.** Children participated in one of four conditions (same story nap, same story no nap, different stories nap, different stories no nap). Children's word recall was tested immediately after reading stories and then children either napped or did not nap. Children's word retention was subsequently tested 2.5 h later, 24 h later, and 7 days later.

Children were tested individually in their normal nursery setting four times within 8 days: immediately after they heard the stories, after a 2.5-h delay (during which time the children who habitually napped took their naps), after a 24-h delay and 7 days after the initial visit, see Figure 1. To increase ecological validity and to allow the children to become familiar and comfortable with the experimenter, she spent a week at the nursery before the experiment helping with routine activities and play (see also Dunn et al., 1977; McLeod and McDade, 2011).

Children were read stories and tested individually in a quiet room (either another classroom or a quiet common area). However, because testing took place in a daycare setting, other people and activities could be sometimes heard, reflecting children's typical daytime shared storybook reading experiences. Note, Riley and McGregor (2012) recently manipulated background noise (quiet, moderate white noise) when novel words were introduced to school-aged children. They tested children's novel word comprehension using 4-alternative forced-choice trials with pictures, as we do in the current study. Importantly for the current study, they found no effect of background noise on children's comprehension for novel names, although they did find an effect on children's word production.

#### Reading phase

During the reading phase, children sat beside the experimenter to ensure the illustrations were easy to see. Children were either read the same story three times or all three different stories once each. Importantly, all children encountered each name-object pair 12 times. Children's questions and comments were neither encouraged nor discouraged (for a similar method see Sénéchal and Cornell, 1993). If the child asked questions the experimenter encouraged the child to return attention to the story (e.g., “let's keep reading and see!”) and avoided naming any objects. The order in which children in the different stories conditions heard the stories was counterbalanced across participants using a Latin Square design. All three stories were read across participants in the same story conditions. Children were given a sticker after each reading to keep them engaged in the task as the nursery/preschool setting is otherwise alluring.

#### Story enjoyment ratings

Children's enjoyment of the stories was examined using a 3-point ratings task (for a similar method rating television programs see Anderson et al., 2000). Immediately after hearing each story, the child was asked to indicate his/her enjoyment of story by giving the experimenter a laminated smiley face card (2′ diameter) from an array. The experimenter asked the child “how much did you enjoy reading this story today?” and set each card on the table, one at a time, explaining what each card represented. For example, “pick this card if you liked the story a lot,” or “pick this card if you didn't like the story.” The order the cards were set on the table was counterbalanced within and across participants, but “a lot” was always placed on the left, “a little” in the middle and “didn't like” on the right. Finally, after hearing all three stories (or after the third reading of the same story), the experimenter asked the child “how much did you enjoy reading all three stories today?”

#### Plot comprehension questions

Immediately after the story enjoyment questions nine plot comprehension questions were administered as an additional control to check children were paying attention to the stories in the different stories conditions. The plot comprehension questions were presented as forced-choice questions and both potential answers were words or phrases that had occurred in the relevant story (to ensure answers appeared in the text, the stories were minimally edited from the originals used by Horst et al., 2011). For example, a question for *Rosie's Bad Baking Day* asked “was Rosie's daddy gone a long time or was he quick?” (He was gone a long time, hence Rosie continues mixing and accidentally uses salt instead of sugar.) A question for *The Very Naughty Puppy* asked “did Rosie pass her mum the book or the phone?” (She handed her mother the phone, so she could arrange for dog obedience classes.) For each child, the correct answers alternated equally often between the first and second choice in the question and whether the answer to Question 1 was first or second was counterbalanced across children. If children answered, “[I] don't know” the experimenter moved on and that question was not included in the child's score (i.e., proportion correct was calculated as the number correct out of the number of questions answered, see Samuelson and Horst, 2007).

We first piloted 12 questions from each story with 12 additional monolingual, British 3-year-old children (5 girls, 7 boys). These children heard each story once and answered all 12 plot comprehension questions immediately after each story. From these questions we selected nine for use in the main study, excluding the easiest and most difficult questions but maintaining the same number of questions per story. There was no difference in difficulty between stories for the questions used in the main experiment, *X*^2^_(16)_ = 3.44, *p* = 0.99 (*M*_baking_ = 0.77, *SD*_baking_ = 0.14; *M*_puppy_ = 0.73, *SD*_puppy_ = 0.21; *M*_restaurant_ = 0.72, *SD*_restaurant_ = 0.27).

Children in the same story conditions were asked nine questions about their story after they had heard it once. Children in the different stories conditions were asked three questions about each story after they had heard the story once (for a total of nine questions). Which questions were asked for a given story was pseudo-randomly determined for each child as questions always occurred in story-chronological order. Plot questions were administered after the story enjoyment ratings so that discussing the plot would not influence children's ratings.

#### Immediate word learning test

The first word learning test occurred immediately after the third story was read and the enjoyment and plot questions were completed. This test included four warm-up trials to ensure the child understood the task. The experimenter told the child that they were going to play “a pointing game” and asked the child to show his or her pointing finger. Then the experimenter opened the test booklet to a practice page and asked the child to indicate each of the pictures in a pseudo-random order (e.g., “can you point to the car?”). Thus, at the end of the warm-up trials the child had practiced pointing to an object in each quadrant (e.g., top left). The same practice page was used for all four trials but different practice pages were used from one test to the next (e.g., +24 h to +7 days). Children were praised for correct choices (100% of trials). Practice page, trial order, and target quadrants were counterbalanced within and across participants.

Next, children's comprehension of the target novel words was tested using the test pages from the test booklet. A different test page was used on each trial. Children were asked to point to each target twice for a total of four test trials. Across trials, targets were presented twice individually and twice together. For example, the child was presented with one *sprock* trial where the *tannin* was also present among the competitors, and one *sprock* trial where the *tannin* was not present among the competitors. Trial order, pages used and quadrant were counterbalanced within and across participants. The word learning task was the same as that used in previous research (Horst et al., 2011; Williams et al., 2011).

#### Delay phase

Working with the staff at the individual nurseries helped ensure that the learning phase was timed to occur no more than 30–45 min before children's regular nap times. After the immediate test, children who habitually napped took their naps and children who did not habitually nap played without any constraints except that they not be read anymore stories until after their next test phase. Children who did not nap were yoked to children who did nap to ensure that there was no difference in the length of the delay phase between groups, see Table 1, *F*_(3, 44)_ = 1.05, *p* = 0.38. There was also no difference in nap length between the same story nap and different stories nap conditions, *t*_(24)_ = 0.44, *p* = 0.67.

**Table 1:** **Delays between the immediate test and post consolidation test, including nap length**.

	**Same story**		**Different stories**	
	**Nap**	**No nap**	**Nap**	**No nap**
	143.33 min	139.00 min	150.00 min	143.00 min
Initial delay	(21.60 min)	(21.15 min)	(18.00 min)	(17.00 min)
	105–170 min	110–175 min	120–165 min	110–170 min
	62.01 min		64.12 min	
Nap length	(8.65 min)		(13.90 min)	
	50–75 min		45–90 min	

Standard deviations presented in parentheses.

#### Subsequent word learning tests (+2.5 h, +24 h, +7 days)

Children were re-tested on their novel name comprehension three more times. The second test occurred approximately 2.5 h after the immediate test, the next occurred approximately 24 h after the immediate test and the final test occurred 7 days after the immediate test (see Figure 1). For each test the same procedure as the immediate test was used.

#### Coding

The experimenter recorded children's responses during each test. A member of the nursery/preschool staff observed the final test for each child to also record responses for inter-coder reliabilities (for a similar method see Horst et al., 2011). Staff members were naïve to the experimental hypotheses and design of the study. Staff members recorded children's responses out of the experimenter's view. Inter-coder reliability was 100%.

## Results

### Word comprehension

**Figure 2:**
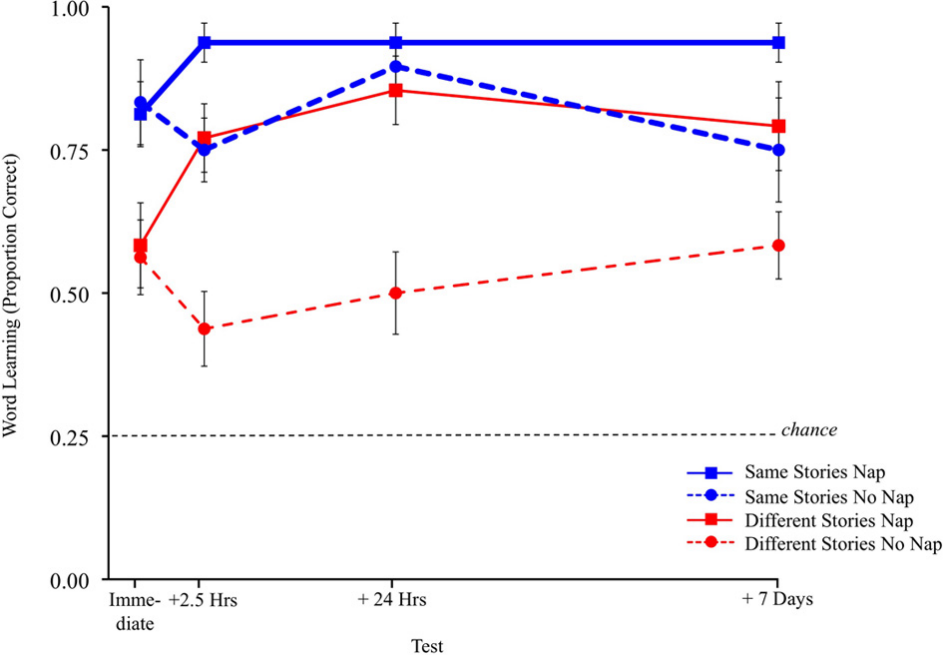
**Children's word learning on each test for each of the four conditions.** Chance is 0.25. Error bars indicate one standard error of the mean.

Here we provide a brief overview of the key findings before delving into the analyses. Results are depicted in Figure 2. As can be clearly seen, children who heard the same story repeated (thin blue lines) learned more words than children who heard different stories (thick red lines), thus replicating previous research. Further, children who napped (solid lines) performed significantly better than children who did not nap (dashed lines). Critically, children who heard different stories but then napped (solid red line), recovered after sleeping and continued to perform just as well as children who had heard the same story repeatedly and did not nap (dotted blue line). In contrast, children who heard different stories and did not nap (dotted red line) never recovered and never performed as well as their peers on the retention tests.

We first present analyses comparing children's word learning against chance and then between conditions. Children's word learning was assessed via 4-alternative forced-choice trials. Overall, children's novel name recall and retention accuracy was significantly better than expected by chance (0.25) for each condition at each test, all *p*s < 0.01 (all of our reported *t*-tests are two-tailed), see Figure 2. However, some of the test alternatives were never-before-seen novel objects (see e.g., Werchan and Gómez, 2014), which may have made the test easier than desired (Axelsson and Horst, 2013b). Recall, half of the trials children received included three novel distractors and half of the trials included the other target as a competitor along with two novel distractors. Presenting items as both targets and non-targets creates a stringent test of word learning (Schafer and Plunkett, 1998; Axelsson and Horst, 2013b). To gain more insight into how well children really learned the target words, we also compared only the trials in which the other target appeared as a distractor to a very conservative level of chance (0.50), see Table 2. When measured in this stringent way, children in the different stories no nap condition failed to demonstrate word learning at any point during the study (all means < 0.50). Children in the different stories nap condition did demonstrate word learning, but only after they had slept. Children in the same story conditions, generally demonstrated significant word learning, as would be expected from previous research (e.g., Horst et al., 2011), with the exceptions that the same story nap condition performed only marginally above chance before their naps (*p* = 0.10) and the same story no nap condition was not performing significantly above chance before overnight sleep (*p* = 0.34) or after 7 days (*p* = 0.27). Note, if chance on these trials is considered 0.25, both same story conditions consistently performed significantly above chance even on these challenging trials (all *p*s < 0.05).

**Table 2:** **Children's responses on word learning trials with the other target as a distractor**.

	**Same story**		**Different stories**	
	**Nap**	**No nap**	**Nap**	**No nap**
Immediate test	0.67^†^	0.79^*^	0.38	0.38
	(0.33)	(0.33)	(0.43)	(0.38)
+2.5 h	0.92^***^	0.58^††^	0.71^*^	0.25^*^
	(0.19)	(0.29)	(0.26)	(0.34)
+24 h	0.92^***^	0.87^**^	0.79^**^	0.25^*^
	(0.19)	(0.23)	(0.26)	(0.34)
+7 days	0.92^***^	0.625^††^	0.75^*^	0.38
	(0.19)	(0.38)	(0.34)	(0.31)

Standard deviations presented in parentheses.

* p < 0.05,

** p < 0.01,

*** p < 0.001 against chance (0.50), note some scores for the different stories no nap group are significantly below chance;

† p < 0.05,

†† p ≤ 0.01 against chance (0.25).

#### Effects of repeated reading and sleep consolidation

Our main interest was the interaction between sleep and story exposure across time. In the following analyses we included data from all of the test trials because including all of the data provides the fullest picture of children's performance (Axelsson and Horst, 2013b), we did run these analyses on only the data from trials where both targets were present and found similar differences between conditions as in the data reported.

To test for differences between sleep and story conditions across time, children's proportions of correct choices were entered into a mixed-design ANOVA with Story Repetition (Same, Different) and Sleep (Nap, No Nap) as between-subjects factors and Test (Immediate, +2.5 h, +24 h, +7 days) as a repeated-measure. The ANOVA yielded a Story Repetition by Sleep by Test Interaction, *F*_(3, 132)_ = 3.24, *p* = 0.02, η^2^_*p*_ = 0.07 (see Figure 2). Thus, story repetition, together with sleep, influences children's word learning across time. The ANOVA also found a Sleep by Test Interaction, *F*_(3, 132)_ = 9.35, *p* < 0.0001, η^2^_*p*_ = 0.18. Sleep continued to influence children's word learning over the course of the study.

Children who heard the same story learned significantly more words than children who heard different stories, *F*_(1, 44)_ = 19.45, *p* < 0.001, η^2^_*p*_ = 0.31, see Figure 2. Further, children who napped learned significantly more words than children who did not nap, *F*_(1, 44)_ = 10.68, *p* = 0.002, η^2^_*p*_ = 0.20. Thus, both stories and sleeping shortly after hearing the stories had a profound effect on children's word learning. Finally, the ANOVA yielded a main effect of Test, *F*_(3, 132)_ = 5.61, *p* = 0.001, η^2^_*p*_ = 0.11. Children performed significantly better after 24 h than immediately after they heard the stories (*p* < 0.001) and than 2.5 h after they heard the stories (*p* < 0.01). Children also performed better 7 days later than immediately after they heard the stories (*p* = 0.01). No other significant effects were found.

#### Tests of simple effects

To better understand how sleep consolidation influences children's word learning via storybooks, we also conducted tests of simple effects. We ran separate ANOVAs for children in the same story conditions and different stories conditions. For children in the same story conditions, proportion of correct choices were entered into a mixed-design ANOVA with Sleep (Nap, No Nap) as a between-subjects factor and Test (Immediate, +2.5 h, +24 h, +7 days) as a repeated-measure. The ANOVA yielded a significant Sleep by Test Interaction, *F*_(3, 66)_ = 4.51, *p* = 0.006, η^2^_*p*_ = 0.17. The ANOVA also yielded a main effect of Test, *F*_(1, 22)_ = 4.51, *p* = 0.05, η^2^_*p*_ = 0.11. Follow-up tests confirmed children performed significantly better after 24 h than immediately after they heard the stories (*p* < 0.01), and than 2.5 h after they heard the stories (*p* < 0.05) and also than 7 days after they heard the stories (*p* < 0.05, see the thin blue lines in Figure 2). No main effect of Sleep was found; however, given that children have done well in previous studies in which they have heard the same stories repeatedly without napping (e.g., Horst et al., 2011), this is not unexpected.

We conducted an identical ANOVA for children in the different stories conditions. The ANOVA yielded a significant Sleep by Test Interaction, *F*_(3, 66)_ = 7.75, *p* < 0.001, η^2^_*p*_ = 0.29. The ANOVA also found a main effect of Sleep, *F*_(1, 22)_ = 8.84, *p* < 0.007, η^2^_*p*_ = 0.55, indicating that children who napped learned significantly more words than children who did not nap. Finally, the ANOVA found a main effect of Test, *F*_(3, 66)_ = 4.11, *p* = 0.009, η^2^_*p*_ = 0.16. Follow-up tests confirmed children performed significantly better 24 h later than immediately after they heard the stories (*p* < 0.01). Children also performed significantly better 7 days later than both immediately after they heard the stories (*p* < 0.01) and than 2.5 h after they heard the stories (*p* = 0.03, see the thick red lines in Figure 2).

### Story enjoyment ratings

Overall, children liked the stories. Only three children answered that they did not like a particular story (one child in the same story no nap condition did not like *Nosy Rosie at the Restaurant;* one child in each of the different stories conditions did not like *Rosie's Bad Baking Day*). A Three-Way Story Repetition × Storybook × Rating contingency test, found no interactions between conditions or stories, all *p*s > 0.32.

All children were asked “How much did you enjoy reading all three stories today?” The majority of children in the same story conditions (83%) answered they liked reading “a lot,” compared to only a third of children in the different stories conditions (33%), confirming that children do enjoy hearing the same stories read repeatedly, see Table 3. This finding is supported by both a Fisher's Exact Test, *p* < 0.001, and an unpaired *t*-test on answers transformed into a 3-point scale as “liked a lot” (3), “liked a little” (2), and “didn't like” (1), *t*_(46)_ = 3.85, *p* < 0.001, *d* = 1.34. Importantly, there was no difference in enjoyment ratings between children who napped and did not nap in the same story conditions, *t*_(22)_ = 0.39, *p* = 0.70, and different stories conditions*, t*_(22)_ = -1.28, *p* = 0.21, suggesting that the word learning differences observed between the two different stories conditions were due to the effect of sleep consolidation and not due to *a priori* differences story enjoyment [in fact, the children who did not nap enjoyed the stories slightly more (*M* = 2.25, *SD* = 0.86) than the children who did nap (*M* = 1.83, *SD* = 0.72)].

**Table 3:** **Children's responses to “How much did you enjoy reading all three stories today?**”

	**Same story**	**Different stories**
“Liked a lot”	20^***^	8
“Liked a little”	3	9
“Did not like”	1	7
Total	24	24

*** p < 0.001, exact binomial test based on p = 0.33 for 20 or more such responses out of 24.

### Plot comprehension

Plot comprehension questions were included as a check to ensure children were attending to the stories during the shared storybook reading episode. Children in the different stories conditions answered three plot questions after each story. Overall, children in the different stories conditions answered the plot comprehension questions at levels significant better than expected by chance [50%, *M* = 0.59, *SD* = 0.11, *t*_(22)_ = 3.14, *p* = 0.005, *d* = 1.34]. Data from two girls (one in each different stories condition) were excluded from these analyses because they scored more than 2.5 standard deviations below (no nap) and above (nap) the means for their conditions. Both children performed similarly to the other children in the conditions on the other tests. There was no effect of story order [*F*_(2, 42)_ = 1.41, *p* = 0.25] or storybook [*F*_(2, 40)_ = 0.55, *p* = 0.58] on plot comprehension scores. Plot comprehension questions were administered before the initial delay phase and there was no difference in performance between children who did and did not nap, *t*_(21)_ = 0.83, *p* = 0.42. Importantly, this again suggests that the word learning differences observed between the two different stories conditions were due to the effect of sleep consolidation and not due to *a priori* differences in story understanding.

Children in the same story conditions answered nine questions about their story after the first reading. Children answered the questions at levels significantly better than expected by chance [50%, *M* = 0.71, *SD* = 0.18, *t*_(23)_ = 7.02, *p* < 0.001, *d* = 1.88], and there was no difference in performance between children who did and did not nap, *t*_(22)_ = 0.19, *p* = 0.85. Data from one child (same story no nap condition) were missing and not included in these analyses (this child performed similarly to the other children in her condition on the other tests). There was no difference in plot comprehension as a function of which storybook children heard [*F*_(2, 21)_ = 0.65, *p* = 0.53]. Note we did not directly compare plot question comprehension performance between story repetition conditions because it was not clear how the methodological differences might influence overall accuracy (e.g., how answering nine questions about the same story might provide scaffolding or how anticipating there will be questions after each new story might have led children to listen more intently).

### Predictive effects of story repetition and sleep

Finally, we conducted a series of multiple regression analyses to determine if Story Repetition (Same, Different), Sleep (Nap, No Nap), Story Enjoyment and/or Plot Comprehension predict children's word learning performance on each retention test. For each regression analysis all predictors were entered simultaneously.

Table 4 depicts the models predicting performance on the first retention test (2.5 h after story exposure). Story Repetition is a significant predictor of word retention [*t*_(47)_ = 3.68, *p* < 0.001, *d* = 1.13] accounting for approximately 23% of the variation in word learning scores. Controlling for Story Repetition, Sleep (napping after story exposure) is also a significant predictor of word retention [*t*_(47)_ = 4.99, *p* < 0.001]. Together, Story Repetition and Sleep account for approximately 50% of the variation in word learning scores [*F*_(2, 47)_ = 22.136, *p* < 0.001, η^2^_*p*_ = 0.33]. Neither Story Enjoyment (*p* = 0.63) nor Plot Comprehension (*p* = 0.65) were significant predictors of word retention 2.5 h after story exposure.

**Table 4:** **A series of regression models predicting children's word retention 2.5 h after story exposure based on story repetition, sleep, story enjoyment and plot comprehension**.

	**Word learning β (standardized)**			
	**Model 1**	**Model 2**	**Model 3**	**Model 4**
Story repetition	0.48^***^	0.48^***^	0.45^***^	0.42^**^
Sleep		0.52^***^	0.52^***^	0.52^***^
Story enjoyment			0.06	0.01
Plot comprehension				0.06
*R*^2^ (adjusted *R*^2^)	0.23 (0.21)	0.50 (0.47)	0.50 (0.46)	0.50 (0.45)

*** p < 0.001,

** p < 0.01.

Table 5 depicts the models predicting performance on the second retention test (24 h after initial story exposure). Again, Story Repetition is a significant predictor of word retention [*t*_(47)_ = 3.74, *p* < 0.001] accounting for approximately 23% of the variation in word learning scores. Controlling for Story Repetition, Sleep is also a significant predictor of word retention [*t*_(47)_ = 3.43, *p* < 0.001]. Together, Story Repetition and Sleep account for approximately 39% of the variation in word learning scores the next day [*F*_(2, 47)_ = 14.50, *p* < 0.001, η^2^_*p*_ = 0.28]. Again, neither Story Enjoyment (*p* = 0.62) nor Plot Comprehension (*p* = 0.43) were significant predictors of word retention 24 h after story exposure.

**Table 5:** **A series of regression models predicting children's word retention 24 h after story exposure based on story repetition, sleep, story enjoyment and plot comprehension**.

	**Word Learning β (standardized)**			
	**Model 1**	**Model 2**	**Model 3**	**Model 4**
Story repetition	0.48^***^	0.48^***^	0.52^***^	0.47^**^
Sleep		0.40^**^	0.39^**^	0.41^**^
Story enjoyment			−0.07	−0.09
Plot comprehension				0.10
*R*^2^ (adjusted *R*^2^)	0.23 (0.22)	0.39 (0.37)	0.40 (0.35)	0.42 (0.36)

*** p < 0.001,

** p < 0.01.

Finally, Table 6 depicts the models predicting performance 7 days later. Story Repetition remains is a significant predictor of word retention [*t*_(47)_ = 2.21, *p* < 0.05], but accounts for much less variation 7 days later than at the earlier time points (approximately 10% of the variation). Again, controlling for Story Repetition, Sleep is also a significant predictor of word retention [*t*_(47)_ = 3.04, *p* < 0.01]. In fact, napping after story exposure is a stronger predictor than Story Repetition. That is, over the long-term, sleep is more beneficial than story repetition for word learning. Together, Story Repetition and Sleep account for approximately 25% of the variation in word learning scores 7 days later [*F*_(2, 47)_ = 7.50, *p* < 0.01, η^2^_*p*_ = 0.20]. Finally, neither Story Enjoyment (*p* = 0.58) nor Plot Comprehension (*p* = 0.39) were significant predictors of word retention 7 days after story exposure.

**Table 6:** **A series of regression models predicting children's word retention 7 days after story exposure based on story repetition, sleep, story enjoyment and plot comprehension**.

	**Word Learning β (standardized)**			
	**Model 1**	**Model 2**	**Model 3**	**Model 4**
Story repetition	0.31^*^	0.31^*^	0.35^*^	0.31^†^
Sleep		0.39^**^	0.38^**^	0.41^**^
Story enjoyment			−0.08	−0.14
Plot comprehension				0.12
*R*^2^ (adjusted *R*^2^)	0.10 (0.08)	0.25 (0.22)	0.26 (0.21)	0.30 (0.22)

** p < 0.01,

* p < 0.05,

† p = 0.05.

Taken together, these data clearly demonstrate that both reading the same story repeatedly and sleeping shortly after story exposure significantly facilitated children's ability to learn words via shared storybook reading. These data also illustrate that sleep consolidation has a profound effect on children's word learning above and beyond story repetition.

## Discussion

Young children enjoy reading storybooks, including reading the same stories repeatedly (Sulzby, 1985; Sénéchal, 1997). The current study replicated previous research demonstrating that repeated readings facilitates word learning via storybooks and extended this research to investigate how sleep consolidation also facilitates word learning in preschool children above and beyond story repetition. Children who either habitually napped or did not nap were either read the same story three times or were read three different stories. Children were tested immediately after the shared reading episode as well as 2.5 h later (after half of the children had napped), 24 h later, and 7 days later. As in previous studies, we found a clear benefit for reading the same story repeatedly (Horst et al., 2011; Williams et al., 2011). Importantly, we also found a clear benefit for sleeping shortly after the shared reading episode. Children who slept after reading the stories performed significantly better than their peers on the later tests.

Most importantly, children who read different stories but then slept performed as well as children who had read the same story repeatedly but had not slept. That is, sleep allowed these children to consolidate the information they had heard such that they could demonstrate later word learning. Learning words from different stories is more difficult than learning words from the same story (e.g., Horst et al., 2011; Williams et al., 2011) and sleep allowed these children to compensate for this more difficult learning situation. In stark contrast, children who heard different stories and did not sleep never caught up to their peers—even after their regular overnight (recovery) sleep. Finally, regression analyses revealed that sleep consolidation was a stronger predictor of long-term word retention than story repetition. Taken together, these data demonstrate clear effects for both repeated readings and sleep consolidation on young children's word learning.

Note, accuracy on the plot comprehension questions did not predict children's word learning at any point during the study. However, the questions we used were designed to provide a check that children were listening to the stories in case we found poor word learning performance. As such, some of these questions tested memory for arguably subtle aspects of the stories (e.g., did Rosie give her mother a phone or a book). Future research should further investigate how comprehension questions can be better designed and used to aid comprehension of both the stories and new words embedded in the stories.

### Relation to storybook reading literature

Previous research has already established that young children learn more words from repeatedly reading the same stories than reading different stories (Horst et al., 2011; Williams et al., 2011; Wilkinson and Houston-Price, 2013) or fewer stories (Sénéchal, 1997; Biemiller and Boote, 2006; McLeod and McDade, 2011). However, independent of our sleep factor, we extend this research in at least two important ways. First, we included intermediate tests of word recall between the initial test (immediately after reading) and the final test (7 days later). A comparison of our two groups who did not nap demonstrates that the advantage for encountering new words from the same storybook is persistent and robust. That word learning performance improves over time is consistent with other word learning studies (Backhaus et al., 2008; Dumay and Gaskell, 2012) and suggests word learning is an extended process (Carey, 1978; Dumay and Gaskell, 2012; McMurray et al., 2012).

Second, after all of the stories were read, we asked all children “how much did you enjoy reading all three stories today?” Over 80% of the children who heard the same story repeatedly answered they liked the shared reading time “a lot,” compared to only 33% of the children who had spent the same amount of time reading, but heard different stories. This has important educational implications because reading for pleasure is related to vocabulary level in later childhood (Sullivan and Brown, 2013). Thus, parents and teachers may want to foster an enjoyment of reading by reading the same stories repeatedly. Indeed, even adults enjoy reading stories when they already know the ending (Leavitt and Christenfeld, 2011).

However, it may be that there are benefits for reading different stories that we have not yet uncovered. Although 3-year-old children often have favorite books—which they repeatedly request—5-year-old children prefer a range of books (Sénéchal, 1988; Sénéchal and LeFevre, 2001). Ultimately, children are likely to learn all they can from a given story and will want to read something new. Research on how children learn words from fast mapping by mutual exclusivity demonstrates that, as in shared storybook reading, children retain words better when the contexts in which they learn the words repeat across learning opportunities (Axelsson and Horst, 2013a; Horst, 2013). However, other research suggests that variability promotes generalization (Perry et al., 2010; Twomey and Horst, 2011; Twomey et al., 2013; Werchan and Gómez, 2014). Thus, one possibility is that repeatedly reading the same story promotes *retention* by supporting children's initial encoding and storage of new name-object associations, but reading different stories facilitates *generalization* by allowing children to extend somewhat familiar name-object associations to new members from the given object category or new situations.

A related possibility is that reading different stories teaches children something about the concepts from the story (in our case object categories) that is not measured with the current tests. For example, in the current study children who heard different stories may have developed a deeper understanding of when and how to use a *sprock*, but were unable to demonstrate this understanding because we did not test them on their memory for the objects' functions or uses (for a similar argument see Biemiller and Boote, 2006). Future research should extend this work and explore how different aspects of shared storybook reading may promote learning of different types of information, e.g., object functions in addition to object names. Such research could also include children's ability to transfer knowledge from pictures to real objects (Ganea et al., 2008, 2011).

### Relation to sleep literature

Previous research demonstrates that sleep supports children's learning (e.g., Hupbach et al., 2009; Henderson et al., 2012; Kurdziel et al., 2013; Wilhelm et al., 2013). The current study adds to this exciting area of research by exploring the word learning benefits of napping in preschool children. Specifically, children who slept after a shared storybook reading episode performed significantly better than their peers on later word learning tests. We know from previous shared storybook reading studies that children's recall for words encountered via different stories is significantly worse than recall for words encountered from the same stories read repeatedly (Horst et al., 2011; Williams et al., 2011; Wilkinson and Houston-Price, 2013), suggesting children's memory traces for words from different stories are weaker. Previously, Diekelmann et al. (2009) have argued that sleep consolidation benefits are greater for weak memory traces than for strong memory traces—which is exactly what we found. Word learning scores were 19% higher when children slept after hearing the same story, but 33% higher when children slept after hearing different stories.

Previous sleep consolidation research has been criticized for design issues (Stickgold, 2013) and circadian effects (Gais et al., 2006), however, the current study does not suffer from these limitations. For example, Stickgold (2013) recently criticized previous research for failing to include a measure of declarative knowledge at the end of training. Specifically, he argues that without such a baseline measure, it is unclear whether sleep is leading to better memory consolidation or whether an equivalent period of wakefulness is leading to forgetting. In the current study, we included a baseline measure (immediate test). A comparison of children's performance on the immediate test and the next test (2.5 h later) indicates that children's performance is both improving after sleep *and* declining after an equivalent similar period of wakefulness.

In addition, several studies test participants in either an AM or PM group (e.g., Gais et al., 2006; Dumay and Gaskell, 2007; Backhaus et al., 2008; Henderson et al., 2012). Unfortunately, in these cases performance may be confounded with time of day. In the current study we tested children at an age where some children still habitually take an afternoon nap and others do not (Mednick, 2013). This allowed us to test all children at the same time of day on each test and avoid circadian confounds (see Lau et al., 2010 for a discussion of the advantages of nap designs). In addition, by re-testing children 1 day (and 1 week) later, we were able to demonstrate that the difference between groups was not due to sleep deprivation because the differences between groups persisted after overnight sleep (for a similar argument see Kurdziel et al., 2013). Finally, we are also able to compare the effect of sleep shortly after learning (nap conditions) to the effect of overnight sleep (no nap conditions). Indeed, our tests of simple effects revealed an important main effect of sleep shortly after learning for children in the different stories conditions. This comparison is possible because we tested children at the same times (see also Hupbach et al., 2009).

However, unlike other studies (e.g., Yoo et al., 2007; Lau et al., 2010) we did not randomly assign children to nap or wake conditions. A major problem for sleep research is that sleep deprivation causes fatigue (Gais et al., 2006), although overnight sleep can control for this (Hupbach et al., 2009; Kurdziel et al., 2013). In the current study, children in the no nap conditions were not deprived of sleep, they had simply given up their naps. Note during early childhood (specifically 3–5 years of age) children who no longer nap sleep for longer at night and therefore children who nap and do not nap sleep for the same amount of time within each 24-h period (Ward et al., 2008; Lam et al., 2011). It is possible that children in the current study who were still habitually napping by 42 months of age had other traits that helped them to learn the words more easily than the children who had given up their afternoon naps. Interestingly, napping may be only beneficial for preschool children who are still habitually having an afternoon nap (Kurdziel et al., 2013). For example, Lam et al. (2011) found 3–5-year-old children who no longer napped performed *better* on an auditory attention span task and vocabulary measures than their same-aged peers who still napped. The authors argue that giving up naps may be a developmental milestone for brain maturation. Critically, in the current study the immediate word learning accuracy scores were no different for children who would go on to nap and not nap in either story condition. Thus, we can be confident that any differences were not present immediately after learning.

Sleep might not be everything. For example, learning across different contexts (as in the case of learning words from different stories) might not benefit from sleep. Recently, Werchan and Gómez (2014) explained that wakefulness aids in forgetting, which is critical for generalization—especially for young children who encode both relevant and irrelevant details. In their study, they taught 30-month-old toddlers names for multiple, distinct exemplars from three novel object categories, which were presented across different contexts (in this case different colored backgrounds). Then, toddlers either napped or remained awake. When tested immediately or 4 h later, only toddlers who had remained awake for several hours generalized the novel object names to novel, never-before-seen exemplars from the object categories. In this case wakefulness facilitated learning, but in the current study sleep facilitated learning—especially for children who heard different stories. Note, however, we only tested children on the original objects from the story (e.g., a yellow *sprock*). Children who heard different stories may have demonstrated better word learning if we had tested them on new exemplars (e.g., a blue *sprock*). Thus, one important difference between the current study and Werchan and Gómez (2014) is whether children were learning to retain or generalize the new object names. Computational modeling work suggests that learning situations that promote later retention may not facilitate generalization and vice versa (Twomey and Horst, 2011; Twomey et al., 2013). Future research is needed to explore the interplay between retention and generalization and how sleep and wakefulness may facilitate different kinds of learning.

### Implications

Due to the constraints of modern society, young children are now sleeping less than ever before and consistently less than recommended guidelines (see Matricciani et al., 2012, for a review). Further, chronically short sleep is significantly related to poorer vocabulary scores (Touchette et al., 2007), childhood obesity (Hart and Jelalian, 2008) and externalizing behaviors, such as tantrums (Scharf et al., 2013).

Many preschool children take an afternoon nap, yet classroom naps are increasingly being curtailed and replaced due to curriculum demands (Kurdziel et al., 2013; Mednick, 2013). Given the growing body of evidence that sleep consolidation has a significant effect on children's learning (e.g., Gais et al., 2006; Gómez et al., 2006; Hupbach et al., 2009; Henderson et al., 2012; Kurdziel et al., 2013; Wilhelm et al., 2013), such policies may be doing our children a huge disservice. In fact, findings like those from the current study indicate we should be encouraging young children to nap and should take advantage of the period right before they nap for instruction in key academic areas such as word learning (Gais et al., 2006) and arithmetic (Lodge, 2013). Kurdziel et al., 2013 even suggest that classroom naps may be particularly beneficial for children with learning delays (for a similar argument see Henderson et al., 2012). In addition, evidence suggests that learning is enhanced during the period *after* sleep (Yoo et al., 2007), suggesting that classroom naps may facilitate learning in the afternoon as well.

## Conclusions

Reading to young children is entertaining (Sénéchal and LeFevre, 2001), provides an opportunity for closeness and bonding (Audet et al., 2008) and helps teach vocabulary (Sénéchal, 1997), print knowledge (Lonigan et al., 2008), and promote later academic abilities (Whitehurst et al., 1988; Rimm-Kaufman and Pianta, 2000). The current study demonstrates that reading to young children before they sleep—as many families do with the routine of the bedtime story (see e.g., Burke et al., 2004)—provides additional benefits in terms of word learning. Without any special training, parents provide children with especially rich language during bedtime story reading, including the kinds of linguistic features that are especially helpful for language acquisition (e.g., repetition, Dunn et al., 1977). Bedtime story reading also does not make the bedtime routine any longer (Field and Hernandez-Reif, 2001). Storybook reading prior to sleep, then, is a relatively quick and easy activity that children both learn from and enjoy: a parent's dream come true.

### Conflict of interest statement

The authors declare that the research was conducted in the absence of any commercial or financial relationships that could be construed as a potential conflict of interest.
